# Patient and healthcare professional perceptions of weight reduction in the treatment of knee osteoarthritis: A systematic literature review

**DOI:** 10.1016/j.ocarto.2026.100875

**Published:** 2026-09-07

**Authors:** Philip G. Conaghan, Catherine Rolland, James Frampton, Louis Lavoie, Katie Giblin, Rebecca Robinson, Magaly Perez-Nieves

**Affiliations:** a Leeds Institute of Rheumatic and Musculoskeletal Medicine, University of Leeds and NIHR Leeds Biomedical Research Centre, Leeds, United Kingdom; b Evidera, a Thermo Fisher Scientific Company, London, United Kingdom; c Evidera, a Thermo Fisher Scientific Company, Montreal, Canada; d Eli Lilly and Company, Indianapolis, IN, 46285, USA

**Keywords:** Patient perspective, Healthcare professionals, Knee osteoarthritis, Systematic literature review, Weight reduction

## Abstract

**Objective:**

Weight reduction confers significant clinical benefits in knee osteoarthritis (OA) management. This systematic review examined patients' and healthcare professionals' (HCPs) perceptions of weight reduction for surgical, pharmacological and non-pharmacological interventions, with emphasis on barriers to implementation.

**Method:**

The review was conducted across Embase, MEDLINE, Cochrane, and PsycInfo for eligible studies using the Population, Intervention, Comparator, Outcome, Timeframe, and Study type framework.

**Results:**

The search identified 2134 publications; of these, 203 had full-text screening, with 46 studies included: 23 qualitative studies, 15 cross-sectional studies, 6 randomized-controlled trials, one quasi-experimental and one observational cohort study. Four main themes of barriers to weight reduction were identified, including limited knowledge, lack of access to interventions, OA-specific barriers to exercise, and non-OA-specific barriers to dieting and exercise. The patient-reported barriers included intrapersonal barriers (i.e., difficulty achieving weight reduction, lack of motivation), physical barriers (i.e., age, challenges with general movement), and social barriers (i.e., lack of social support). The HCP-reported barriers included knowledge barriers, communication barriers, and systemic barriers (i.e., time constraints and resource limitations). Both patients and HCPs had varying expectations and satisfaction levels regarding the impact and delivery of weight reduction interventions**.**

**Conclusions:**

Despite broad recognition of weight reduction's benefits in knee OA, substantial barriers hinder adherence and implementation. Targeted strategies addressing patient-HCP communication, alongside enhanced education and training for both groups, are critical to improving weight reduction outcomes in this population. There is currently a gap in evidence around barriers to gastric surgery and pharmacological treatment specific to patients with knee OA.

## 1 Introduction

Osteoarthritis (OA) is the most common type of arthritis, and the knee is the most frequently affected joint [1]. Knee OA (KOA) impacts 528 million people worldwide [1]. The prevalence of OA is rising because of population aging and increasing rates of obesity [2]. Although genetics and aging are significant risk factors, weight reduction can delay the onset, slow the progression, and decrease the severity of KOA in terms of reduced pain and improved physical function [3,4].

Evidence-based KOA treatment guidelines recommend a multimodal approach to reduce pain, improve function, and enhance the overall quality of life of affected individuals [[5], [6], [7]]. Nonpharmacological interventions include patient education, exercise, and weight reduction. Exercise aims to strengthen surrounding muscles, increase joint stability, promote mobility, and reduce the load placed through the knee. Pharmacological options, including analgesics and nonsteroidal anti-inflammatory drugs, are prescribed primarily to manage pain [7]. In severe cases, surgical interventions, such as joint replacement, may be considered [7].

Weight reduction is a commonly recommended intervention for KOA supported by clinical guidelines, healthcare practices, and public health initiatives [[5], [6], [7], [8]]. Weight reduction benefits multiple pathways involving OA symptoms and progression [9,10]. Beyond KOA improvements, weight reduction ≥10% improves quality of life and lowers the risk or severity of comorbidities, such as cancer, diabetes mellitus, hypertension, and sleep apnea [11]. However, evidence of effective utilization of weight reduction for KOA is limited. Even within randomized controlled trials (RCTs), a 42% attrition rate was observed at 1-year follow-up in a trial investigating the impact of weight reduction on KOA symptoms [12].

With the advent of new pharmacologic treatments for overweight and obesity, there is a need to understand patients' and physicians' perceptions of weight reduction in the treatment of KOA. Although systematic literature reviews (SLRs) of patients' and healthcare professionals’ (HCPs) perceptions of lifestyle changes to reduce the burden of KOA have been reported [[13], [14], [15]], corresponding evidence of perceptions of weight reduction achieved by any interventions, including pharmacological and surgical approaches, is lacking.

Therefore, a comprehensive SLR was conducted to better understand patient and HCP perceptions of surgical, pharmacological and non-pharmacological weight reduction in the treatment of KOA, with a main focus on the identified barriers to weight loss in people with KOA.

## 2 Methods

### 2.1 Literature search

This SLR was conducted according to the Preferred Reporting Items for Systematic Reviews and Meta-analyses [16] and the *Cochrane Handbook for Systematic Reviews of Interventions* [17] and was registered in PROSPERO (CRD42023420014). Embase, MEDLINE, the Cochrane Central Register of Controlled Trials, the Cochrane Database of Systematic Reviews, and PsycINFO databases were searched to capture peer-reviewed articles published in English from January 1, 2010, through November 17, 2025. Search terms included a combination of free text and controlled vocabulary terms for the population, interventions, and study design filters (Supplementary Table 1). Bibliographies of relevant SLRs identified in the electronic database search were screened for publications that were not identified in the systematic search.

### 2.2 Study selection

Each study was assessed against eligibility criteria structured according to the population, intervention, comparator, outcome, and study design (PICOS) framework (Table 1) systematically using a two-step process. Dual reviewer screening was performed using DistillerSR (Version 2.35. DistillerSR Inc.; 2023). First, the title and abstract of each publication were screened independently by two reviewers (JF and LL) to determine the publication's eligibility for inclusion according to the criteria described in the PICOS table. Next, the full-text articles from the included abstracts were screened by two independent reviewers (JF and LL), again, according to the criteria in the PICOS table. Any disagreements were resolved by a third investigator (CR).

**Table 1:** Inclusion and exclusion criteria.

Study	Inclusion criteria	Exclusion criteria
Patient population	Patients with overweight/obesity and KOA HCPs managing patients with overweight/obesity and KOA	Patients with other forms of OA, mixed populations for which no data specific for KOA are available; and pregnant or lactating patients
Intervention - Treatment	Weight reduction (i.e., diet, exercise, pharmacological treatment, or bariatric surgery)	Alternative weight-reduction approaches (i.e., herbal supplements, hypnosis, or acupuncture)
Intervention - Comparison	No restrictions	NA
Outcomes	Barriers to successful weight reduction HCP or patient perceptions/expectations of weight reduction and KOA symptom reductions Satisfaction with current approaches Psychological impact of obesity/overweight on KOA Frequency of targeting weight reduction in patients with KOA Methods used to achieve weight reduction and change in weight in response to treatment Primary manager and other HCPs involved in weight reduction (i.e., general practitioner, orthopaedist, rheumatologist, physiotherapist, lifestyle coach)	• Clinical outcomes for efficacy and safety of treatment • Outcomes not related to HCP approach to weight reduction or patients' perception of weight reduction
Study design	Clinical trials (perception outcomes only) and observational studies, including surveys, interviews, questionnaires, focus groups, patient preference studies, and systematic reviews and meta-analyses (for citation chasing only)	Case reports; economic, pharmacodynamic, genetic, cellular, molecular, or animal studies; dissertations; letters, editorials, comments, notes, and erratum; trial protocols; and non-systematic reviews

Abbreviations: HCP = healthcare professional; KOA = osteoarthritis of the knee; NA = not applicable; OA = osteoarthritis.

### 2.3 Data extraction

Data extraction grids were developed to extract the data elements of interest. Publication details, study characteristics, population characteristics, intervention details, and patient and HCP perspectives were extracted by one investigator (JF). Validation was performed by a second investigator (LL). Any disagreements were resolved through discussion with a third investigator (CR). For qualitative studies, themes, subthemes and associated quotes that addressed the outcomes of interest were extracted. For semi-quantitative studies (e.g., satisfied vs unsatisfied, different levels of satisfaction), proportions of patients (n, %) were extracted.

### 2.4 Synthesis of the findings

The authors performed a thematic analysis of the qualitative and semi-quantitative data informed by previously published methods [18]. This involved familiarization with the data and extraction of quotes which were relevant to the outcomes of interest. Initial themes were established by authors and were iteratively reviewed. A process of interrater reliability was used to establish themes based mainly on a reflexive and interpretivist approach. Assumptions, definitions and decisions were discussed at each iteration. Themes were refined to ensure the data set selected were considered in the correct theme to allow for a meaningful interpretation of the findings.

### 2.5 Study quality and bias assessment

Study quality was assessed using the Critical Appraisal Skills Programme for qualitative studies [19], the Newcastle-Ottawa Scale for observational longitudinal, quasi-experimental and cross-sectional studies [20] and the Cochrane Risk-of-Bias Assessment Tool 2.0 for RCTs [21].

## 3 Results

### 3.1 Search results

**Fig. 1:**
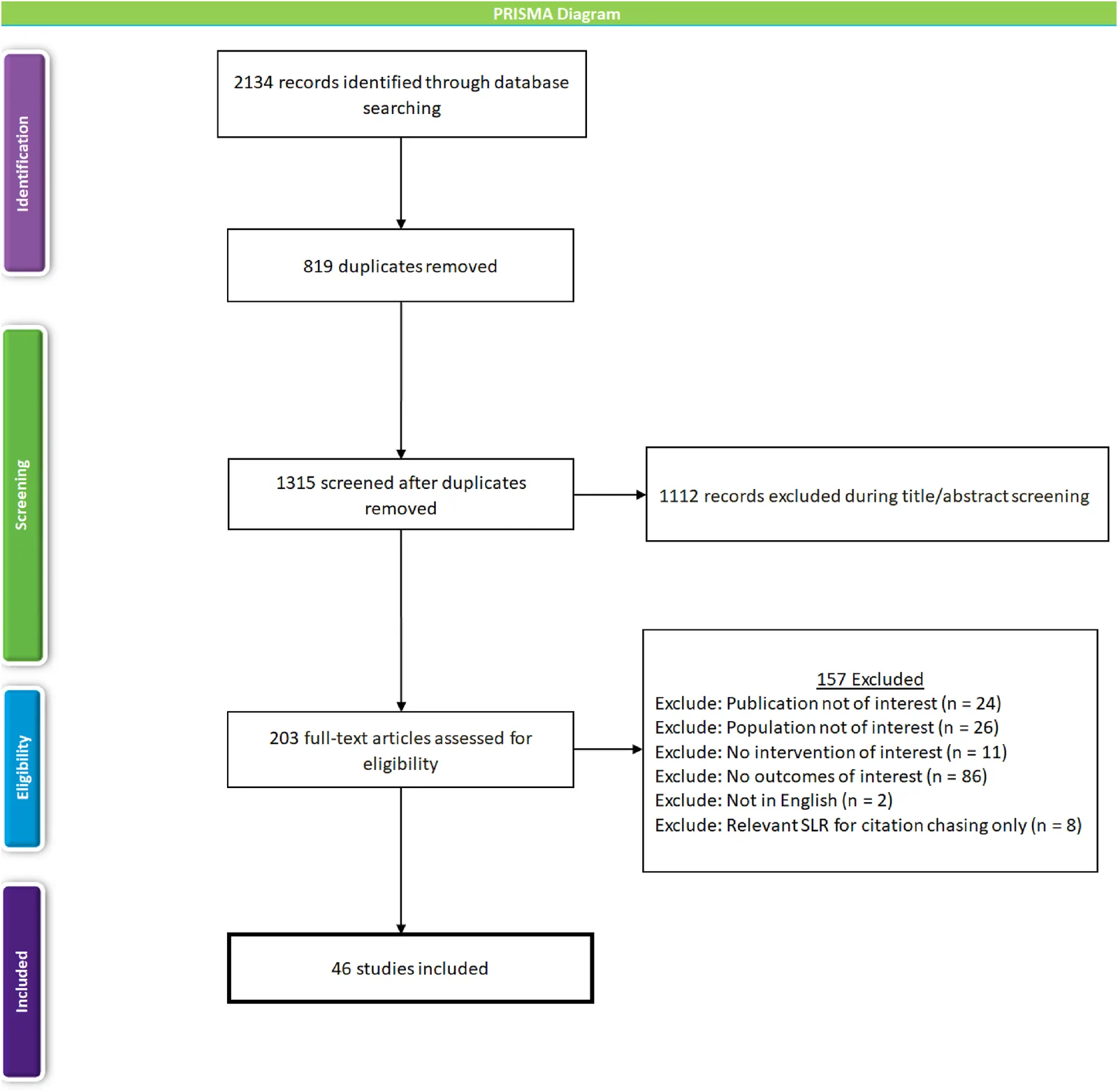
Preferred Reporting Items for Systematic Reviews and Meta-analyses flow diagram.

The literature search yielded 2134 publications. After removing duplicates, 1315 unique publications were considered for title and abstract screening. In total, 203 articles were assessed at the full-text level; of these, 157 publications were excluded. A total of 46 publications were included in the SLR (Fig. 1).

### 3.2 Study characteristics

Among the included studies, 23 were qualitative [[22], [23], [24], [25], [26], [27], [28], [29], [30], [31], [32], [33], [34], [35], [36], [37], [38], [39], [40], [41], [42], [43], [44]], 15 were cross-sectional observational [[45], [46], [47], [48], [49], [50], [51], [52], [53], [54], [55], [56], [57], [58], [59]], six were RCTs [[60], [61], [62], [63], [64], [65]], one was a quasi-experimental [66] and one was an observational cohort [67]. Three of the six RCTs only reported the psychological impact of obesity numerically [61,62,65], and one RCT only reported that more participants receiving a very low energy diet with exercise were satisfied with their programme compared to those in an exercise only control group [60]. Hence, only two of the six RCTs [63,64] are considered in detail in this SLR.

Studies were conducted across Australia, Europe, North America, and Asia between 2004 and 2025. The included studies investigated patients' perceptions (n = 28) [22,[25], [26], [27],^31^,^33^,^34^,^39^,^40^,^42^,^43^,^45^,^46^,^48^,^49^,^53^,^54^,[56], [57], [58], [59], [60], [61], [62], [63],[65], [66], [67]], HCPs' perceptions (n = 11) [22,23,[28], [29], [30],[36], [37], [38],^50^,^51^,^55^], or both patients' and HCPs’ perceptions (n = 7) [32,35,41,44,47,52,64]. The full details of the study characteristics are provided in Table 2. No studies reporting barriers to surgical and pharmacological weight loss in patients with KOA were identified.

**Table 2:** Study characteristics.

Author, year	Study design	Data source	Number of centers	KOA definition	Inclusion/Exclusion criteria	Period (years)	Country (sponsor)
**Studies investigating patient perceptions**							
Allison et al. [22]	Semi-structured telephone interview	Primary research	NR	NICE diagnostic criteria for clinical OA [68]; patient questionnaire; knee only	Inclusion criteria: Diagnostic criteria for clinical OA [68] and (1) age ≥45 years; (2) activity-related knee joint pain; (3) no morning knee joint-related stiffness or stiffness lasting <30 min; and a BMI in the overweight or obese categories (>25 kg/m^2^) Exclusion criteria: NR	2018	Australia (University of Melbourne)
Allison et al. [60]	RCT	Primary research	Multicenter	NICE diagnostic criteria for clinical OA [68]	Inclusion criteria: (1) meet NICE clinical criteria for OA; (2) aged ≥45 years; (3) activity-related knee joint pain; (4) morning knee stiffness ≤30 min; (5) knee pain ≥3 months; (6) knee pain on most days of past month; (7) knee pain ≥4/10 during walking in past week; (8) BMI >27 kg/m^2^; (9) if on antihypertensive medication, willing to check blood pressure if light-headed/dizzy Exclusion criteria: (1) weight >150 kg; (2) on waiting list for or planning knee/hip or bariatric surgery in next 6 months; (3) knee surgery on affected knee in past 6 months; (4) self-reported inflammatory arthritis; (5) weight loss >2 kg in past 3 months; (6) actively trying to lose weight (meal replacements, slimming clubs, professional support, weight-loss drugs, structured meal programs); (7) unwilling to maintain current diet if randomized to exercise-only group; (8) unable to undertake ketogenic VLCD due to: Type 1 diabetes, type 2 diabetes requiring insulin/other meds (except metformin), warfarin use, stroke/cardiac event in past 6 months, unstable cardiovascular conditions, fluid-intake restrictions, renal issues without GP clearance (eGFR >30 mL/min/1.73m^2^), neurological lower-limb conditions, or vegan diet requirements.	2021 to 2023	Australia (University of Melbourne)
Aree-Ue et al. [66]	Quasi-experimental study	Primary research	NR	KOA classified by ACR criteria	Inclusion criteria: (1) mild to moderate KOA classified by the ACR criteria; (2) BMI of 23 kg/m^2^ (using asian criteria for BMI classified as overweight and pre-obese); and (3) no cognitive impairment Exclusion criteria: (1) secondary KOA (i.e., history of knee injury or rheumatoid arthritis); (2) history of knee surgery; (3) any injections in either knee 3 months before study participation; (4) serious medical conditions, such as myocardial infarction; and (5) progressive symptoms or severe KOA requiring surgery.	NR	Thailand (NR)
Baumbach et al. [45]	Cross-sectional study	Clinic EMR	Single center	ICPC-2-R code of L90 (KOA), L91 (OA) in the knee, or a recurrent L15 (knee complaint)	Inclusion criteria: (1) age ≥30 years; (2) first or follow-up KOA consultation between September 1, 2017 and February 28, 2019; and (3) an ICPC-2-r code of L90 (KOA), L91 (OA) in combination with the word “knee” mentioned in free text, or a recurrent L15 (knee complaint) with no adequate trauma or other explanation Exclusion criteria: NR	2018 to 2019	Denmark (EIT Health)
Bennell et al. [61]	RCT	Primary research	Single center	NICE diagnostic criteria for clinical OA [68]	Inclusion criteria: Diagnostic criteria for clinical OA [68] (no required radiographic evidence) and (1) an age of 45–80 years; (2) knee pain most days for at least 3 months; (3) average knee pain severity of at least 4 on an 11-point NRS in the previous week (0 = no pain to 10 = worst pain possible); and a BMI ≥28 kg/m^2^ and <41 kg/m^2^ Exclusion criteria: (1) conditions requiring medical monitoring for dietary interventions, including a BMI ≥41 kg/m^2^, type 1 diabetes, type 2 diabetes requiring medication (except for metformin), warfarin use, a stroke or cardiac event in the past 6 months, unstable heart condition, and fluid intake restriction; (2) inflammatory arthritis; and (3) knee surgery within the past 6 months	2018 to 2021	Australia (Medibank, the medibank better health foundation research fund, and a national health and medical research council center of research Excellence).
Bopf et al. [46]	Cross-sectional study	Ipswich general hospital (Queensland) orthopedic department outpatient waiting list	Single center	GP referral with a provisional diagnosis of KOA	Inclusion criteria: (1) age >50 years and (2) no pre-existing diagnosis of inflammatory disease. Patients were only included as bilateral cases if they could not identify “the worst” side Exclusion criteria: NR	NR	Australia (NR)
Bunzli et al. [25]	Qualitative interviews	St Vincent's hospital (melbourne)	Single center	KOA diagnosis (end-stage KOA on the waiting list for TKA)	Inclusion criteria: (1) age ≥18 years; (2) English speakers; (3) a diagnosis of KOA; and (4) consent for primary TKA and on the waiting list. Exclusion criteria: (1) patients requiring an interpreter and (2) inability to provide independent informed consent for TKA due to cognitive impairment	2018	Australia (NR)
Carmona-teres et al. [26]	Interpretative qualitative study	Primary research	NR	NR	Inclusion criteria: (1) KOA diagnosis and (2) residence in the metropolitan area of barcelona Exclusion criteria: NR	2015	Spain (The carlos III institute of health ministry of economy and competitiveness, european union ERDF)
Coetzee et al. [27]	Semi-structured in-depth individual interviews	Primary research	NR	KOA diagnosis	Inclusion criteria (1) KOA diagnosis by physician, registered nurse, or physiotherapist and (2) age ≥45 years Exclusion criteria: (1) other conditions that could impact functional abilities (i.e., stroke, gout, or rheumatoid arthritis) and (2) unclear diagnosis	2018	South Africa (National research Foundation)
Ekram et al. [48]	Cross-sectional study	Primary research	Single center	NR	Inclusion criteria: (1) age 50–70 years and (2) symptomatic KOA Exclusion criteria: (1) inability to provide informed consent due to inability to speak or read English	2011 to 2012	Australia (NR)
Grønhaug et al. [49]	Cross-sectional study	Primary research	Multicenter	Radiological diagnosis of OA or signs or symptoms of OA	Inclusion criteria: (1) OA diagnosis with ICPC codes of L89 (OA of the hip), L90 (KOA) or L91 (OA, other) or clinical signs and symptoms corresponding to OA in the hip or knee Exclusion criteria: Inflammatory rheumatic diseases, malignant illness, or other conditions considered by the GP to affect the ability to complete the questionnaire	2012	Norway (NR)
Howarth et al. [53]	Cross-sectional questionnaire study	Primary research	Single center	NR	Inclusion criteria: (1) BMI ≥30 kg/m^2^; (2) radiographic changes in KOA (either new or review); and (3) ability to understand written English Exclusion criteria: Other forms of arthritis	NR	UK (NR)
Ingelsrud et al. [54]	Cross-sectional study	Primary research	Single center	NR	Inclusion criteria: Referral for first-time appointments with an orthopedic surgeon for an assessment of KOA Exclusion criteria: (1) inability to speak or read Danish and (2) cancellation of consultation	2018 to 2019	Denmark (The Danish rheumatism association and by the orthopedic department at the Copenhagen University hospital Hvidovre)
Isla pera et al. [31]	A phenomenological qualitative study	Therapeutic education and functional readaptation (TEFR) program	NR	NR	Inclusion criteria: Elderly persons with KOA Exclusion criteria: NR	2013	Spain (NR)
Lawford et al. [33]	Qualitative patient interview	Secondary research	NR	NICE OA clinical criteria	Inclusion criteria: (1) age 45–80 years with a clinical diagnosis of knee osteoarthritis per NICE and Australian clinical care standard guidelines; (2) assigned to the weight-loss arm of the 6-month RCT (ACTRN12618000930280) (3) reports activity-related knee joint pain; (4) morning knee stiffness ≤30 min; (5) knee pain on most days for ≥3 months; (6) average knee pain in the past week ≥4/10 on an 11-point NRS (7) BMI 28–40.9 kg/m^2^; (8) medibank private member with arthroplasty-eligible cover; (9) willing to follow self- management advice, engage in exercise/physical activity, and/or participate in the weight-loss program; (10) able to regularly self-weigh (e.g., access to bathroom scales) Exclusion criteria: (1) booked for knee surgery on either knee; (2) all eligible knee joints already replaced (bilateral replacements or unilateral replacement with non-operated knee pain <4/10); (3) knee surgery within the past 6 months; (4) self-reported rheumatoid arthritis or other inflammatory arthritis; (5) any medical condition or upcoming procedure that, in the opinion of research staff/investigators, would preclude participation (6) for participants identified as at risk from pre-exercise or falls screening, GP does not provide clearance; (7) use of low-calorie meal replacement products in the past 6 months; (8) currently, or within the past 6 months, undertaking regular knee strengthening exercise; (9) unable to undertake a very low energy/Ketogenic diet for medical reasons	NR	Australia (medibank; medibank better health foundation research fund; national health and medical research council (NHMRC) center of research excellence)
Lawford et al. [34]	Semi-structured interview following from RCT	Secondary research	Multicenter	NICE OA clinical criteria	Inclusion criteria: (1) participation in the POWER RCT trial (NCT04733053); (2) diagnosis of knee OA using the national institute for health and care excellence clinical OA criteria; (3) history of knee pain ≥3 months, knee pain on most days of the past month, and knee pain during walking over the past week of at least 4 on an 11-point numeric rating scale; (4) body mass index (BMI) greater than 27; (5) willingness to monitor blood pressure if using hypertensive medication and light-headed/dizzy during the trial; (6) ability to give informed consent and participate fully in trial procedures Exclusions criteria: (1) aged >80 years; (2) weigh >150 kgs; (3) on waiting list for/planning knee/hip surgery or bariatric surgery in next 6 months; (4) previous arthroplasty or recent surgery (past 6 months) on affected knee; (5) inflammatory arthritis (e.g., rheumatoid arthritis); (6) weight loss more than 2 kg over the past 3 months; (7) actively trying to lose weight; (8) unwilling to continue current dietary patterns if randomized to the exercise group; conditions in which the dietary intervention necessitated medical monitoring (type 1 diabetes); (9) type 2 diabetes requiring medication except metformin); (10) warfarin use; (11) stroke/cardiac event in past 6 months; (12) renal problems (unless clearance obtained from general practitioner and estimated glomerular filtration rate >30 mL/min/1.73m2); (13) fluid intake restriction; (14) unstable cardiovascular condition; (15) neurologic condition affecting lower limbs; (16) vegan dietary requirements (because of complexity of delivering a nutritionally complete diet with the dietary intervention); (17) unable to speak English	NR	Australia (medibank; medibank better health foundation research fund; national health and medical research council (NHMRC) center of research excellence)
Messier et al., 2025 [62]	RCT	Primary research	Multicenter	American college of rheumatology (ACR) clinical criteria for knee OA	Inclusion criteria: (1) ambulatory, community-dwelling adults with BMI ≥27 kg/m^2^; (2) meet ACR clinical criteria for knee OA; (3) age ≥50 years; (4) knee pain most days plus ≥2 of: stiffness <30 min, crepitus, bony tenderness, bony enlargement, or no palpable warmth Exclusion criteria: (1) symptomatic coronary artery disease; (2) type 1 diabetes; (3) BMI <27 kg/m^2^; (4) does not meet ACR clinical criteria for knee OA	2016 to 2019	USA (National Institute of Arthritis and Musculoskeletal and Skin Diseases of the NIH (grant 1-u01-ar-068658-01))
Pavlovic et al., 2023 [39]	Qualitative patient interview	Primary research	Multicenter	NR	Inclusion criteria: (1) adults with end-stage osteoarthritis (2) BMI ≥30 kg/m2 (3) Waitlisted for elective primary total hip or knee replacement surgery Exclusion criteria: (1) Non-English or Arabic speakers (2) Had surgery brought forward (3) Had medical conditions limiting adherence to the diet (4) Were fasting for religious or medical reasons or were already participating in a weight-loss diet (intervention cohort only).	Control group: July to September 2019 Intervention group: October 2019 to February 2020	Australian and New Zealand (musculoskeletal (ANZMUSC) clinical trials network and medibank better health foundation (MBHF))
Østerås et al. [57]	Cross-sectional study	Primary research	Multicenter	KOA diagnosis	Inclusion criteria: (1) KOA diagnosis and (2) age ≥45 years Exclusion criteria: (1) inflammatory arthritis; (2) malignant illness; and (3) inability to complete the questionnaire	2012 to 2014	International (EULAR health professional Grant)
Oomen et al. [56]	Cross-sectional study	Primary research	NR	Clinical KOA according to ACR criteria	Inclusion criteria: (1) ACR KOA classification criteria; (2) Dutch language proficiency; (3) residence in The Netherlands; and (4) age ≥40 years Exclusion criteria: None	2019	The Netherlands (NR)
Richette et al. [58]	Cross-sectional study	Primary research	Multicenter	Symptomatic KOA according to ACR criteria	Inclusion criteria: (1) KOA diagnosis; (2) age ≥18 years; and (3) and no other conditions that could interfere with the assessment of KOA Exclusion criteria: NR	2008	France (Wyeth-santé familiale France)
Seward et al. [63]	RCT	Primary research	Multicenter	Radiographically confirmed arthritis	Inclusion criteria: (1) ≥18 years; (2) BMI 40–47 kg/m^2^; (3) device compatible with video calls; (4) indicated for primary unilateral TJA; (5) radiographic arthritis with functional limitations after failed nonoperative care. Exclusion criteria: (1) planned bariatric surgery in the next 6 months; (2) BMI >47 kg/m^2^.	2019 to 2023	USA (vela foundation)
Siddique et al. [59]	Cross-sectional study	Primary research	Single center	ACR criteria	Inclusion criteria: (1) women aged ≥40 years; (2) clinically diagnosed knee OA per ACR criteria, supported by WOMAC; (3) residing in selected areas of kannur for ≥1 year. Exclusion criteria: NR	2024	India (NR)
Somers et al. [65]	RCT	Primary research	Single center	ACR criteria for OA and radiographic evidence of OA	Inclusion criteria: (1) knee pain on most days of the month for at least the prior 6 months; (2) age >18 years; (3) BMI classified as overweight or obese (BMI ≥25 and ≤ 42); (4) ACR criteria for OA and radiographic evidence of OA affecting one or both knees (i.e., knee x-rays grades based on the kellgren-lawrence grading system (0–4)); (5) no other major weight-bearing joint affected by OA; (6) HCP assessment of KOA as the medical condition contributing the most to limitations in daily function; and (7) ability to read and speak English Exclusion criteria: (1) medical conditions that increase the risk of a significant adverse health event during physical activity (e.g., myocardial infarction in the previous 6 months or abnormal blood pressure response to exercise); (2) another known organic disease that contraindicates safe participation in the study (e.g., cancer); (3) a non-OA inflammatory arthropathy or another arthritic disorder (e.g., rheumatoid arthritis); (4) regular use of oral corticosteroids; and (5) participation in a regular exercise or weight-reduction program	2004 to 2008	USA (NR)
Singh et al. [40]	Nominal group technique sessions	Primary research	Single center	ICD-10-CM code M17.x for KOA	Inclusion criteria: (1) at least one outpatient visit at a community-based outpatient clinic and (2) an ICD-10-cm code M17.x for KOA Exclusion criteria: NR	2020	USA (University of Alabama)
Tan et al. [42]	Survey questionnaire	Primary research	Multicenter	NICE clinical diagnostic criteria for knee OA	Inclusion criteria: (1) independent community mobilizers; (2) meet NICE clinical diagnostic criteria for knee OA. Exclusion criteria: (1) alternate diagnosis of knee OA; (2) other forms of knee arthritis (inflammatory or post-traumatic); (3) moderate–severe cognitive impairment; (4) previous knee arthroplasty; (5) wheelchair-bound; (6) medical conditions that interfere with rehabilitation (e.g., decompensated heart failure, significant post-stroke deficits, end-stage renal failure).	2021 to 2022	Singapore (None)
Toye et al. [43]	In-depth interview	Primary research	Single center	NR	Inclusion criteria: NR Exclusion criteria: NR	NR	UK (NR)
Vincent et al. [67]	Observational study	Osteoarthritis initiative (OAI) clinical dataset version 0.2.3	Multicenter	Radiographic imaging	Inclusion criteria: Patients with knee OA in the OAI clinical data set Exclusion criteria: NR	NR	USA (none)
**Studies investigating HCP perceptions**							
Allison et al. [23]	Qualitative healthcare provider interview	Secondary research	Single center	Chronic (≥3 months) knee pain and a clinical diagnosis of knee OA as per NICE criteria	Inclusion criteria: (1) participation in the POWER RCT (NCT04733053); (2) musculoskeletal physiotherapists registered with AHPRA and delivering interventions in both trial arms (VLED + exercise and exercise-only); (3) working in private practice in Victoria and recruited via clinician networks and APA advertisements. Exclusion criteria: NR	NR	Australia (university of melbourne)
Allison et al. [24]	Qualitative semi structured telephone interview	Primary research	NR	NR	Inclusion criteria: (1) physical therapists currently registered with the Australian Health Practitioner Registration Agency; (2) current practice in Australia; and (3) treated at least 1 patient with KOA within the past 12 months Exclusion criteria: NR	2017	Australia (Early career seeding grant by the university of Melbourne School of health Sciences)
Egerton et al. [28]	Semistructured interviews	Primary research	NR	NR	Inclusion criteria: (1) GPs practicing in a primary care setting and (2) treated at least one patient with KOA per month. Exclusion criteria: NR	NR	Australia (National health & medical research council center of research excellence in translational research in musculoskeletal Pain)
Ferreira et al. [29]	Concurrent mixed-methods (e-survey and semi-structure interviews)	Primary research	NR	NR	Inclusion criteria: (1) Portuguese physical therapists (2) Member of the Portuguese national physical therapy professional association (APFISIO) Exclusion criteria: NR	NR	Portugal (NR)
Godziuk et al. [30]	Qualitative survey	Primary research	NR	NR	Inclusion criteria: (1) orthopedic surgeons (2) Perform hip and/or knee arthroplasty in the USA Exclusion criteria: NR	2022	USA (NR)
MacKay et al. [36]	Qualitative semistructured interview	Primary research	NR	NR	Inclusion criteria: (1) physical therapists registered with their provincial regulatory body and (1) treated individuals with knee symptoms (i.e., pain, aching, and stiffness) or diagnosed KOA in the past 3 months; (2) practice in community-based or outpatient settings (e.g., private clinics, primary care practice, and hospital outpatient departments); and (3) ability to communicate in English Exclusion criteria: NR	NR	Canada (NR)
Hill et al. [51]	Cross-sectional study	Primary research	NR	NR	Inclusion criteria: (1) consultant members of BASK; (2) specialist practice in knee surgery; and (3) have been proposed and seconded by current society members to meet membership criteria Exclusion criteria: Did not currently undertake knee arthroplasty surgery in the UK	2017	UK (Wrightington lower limb unit research fund, John Charnley Trust)
Hill et al. [50]	Cross-sectional study	Primary research	NR	NR	Inclusion criteria: Members of specialty interest groups within the Royal College of General Practitioners Exclusion criteria: NR	NR	UK (John Charnley Trust)
Mal et al. [55]	Cross-sectional survey	Primary research	NR	NR	Inclusion criteria: (1) physical therapists practicing in Saudi Arabia; (2) managed at least one patient with knee OA in the past 6 months. Exclusion criteria: (1) non-physiotherapist healthcare professionals; (2) undergraduate students; (3) individuals not practicing in Saudi Arabia.	NR	Saudi Arabia (taif university)
Nguyen et al. [38]	Semi-structured interviews embedded in a RCT	Primary research	Multicenter	NR	Inclusion criteria: Private practice physiotherapists located across metropolitan sydney Exclusion criteria: NR	NR	Australia (none)
**Studies investigating patient and HCP perceptions**							
Lawford et al. [35]	Qualitative semi structured telephone interview	Secondary research	NR	NICE OA clinical criteria	Inclusion criteria: (1) patients randomized to the weight-management arm of the trial who completed the 6-month intervention; (2) private health insurance with medibank private at a level that included coverage for arthroplasty surgery; (3) NICE OA clinical criteria; (4) average knee pain ≥4 on an 11-point numeric rating scale (0 = no pain, 10 = worst pain possible) in the past week; (5) a history of knee pain on most days for ≥3 months; (6) age <81 years; and (7) BMI from 28 kg/m^2^ to <41 kg/m^2^ Exclusion criteria: NR	2019 to 2020	Australia (Medibank and the medibank better health foundation research fund, national health and medical research council center of research Excellence)
Wallis et al. [44]	Online survey	Primary research	NR	NR	Inclusion criteria: *Patients*: (1) patient age >40 years and (2) hip or knee pain with or without a previous diagnosis of hip OA or KOA *HCPs*: (1) registered orthopedic surgeons, sports physicians, rehabilitation physicians, rheumatologists, or GPs and (2) currently involved in managing patients with hip OA and/or KOA. Exclusion criteria: NR	NR	Australia (The cabrini Foundation)
Knoop et al. [32]	Qualitative semistructured interviews	Primary research	Single center	NR	Inclusion criteria: *Patients*: (1) knee pain (NRS during walking ≥2/10) persisting for at least 3 months as their reason to visit the PT; (2) a clinical diagnosis of KOA according to the ACR criteria; and (3) informed consent *HCPs:* (1) physiotherapists and dieticians and (2) treated at least three or two patients Exclusion criteria: *Patients*: (1) age <40 or >85 years; (2) severe knee pain (i.e., NRS pain severity during walking ≥9/10); (3) physical or mental comorbidity severely affecting daily life and a contraindication for the usage of exercise therapy; (4) suspicion of chronic widespread pain (i.e., pain present for at least 3 months in at least three joints; (5) TKA or on a waiting list for TKA; and (6) knee pain for reasons other than KOA (e.g., rheumatoid arthritis and gout); (7) PT or intra-articular injections in the past 6 months for knee pain; and (8) insufficient comprehension of Dutch language		The Netherlands (Scientific board physical therapy of the royal Dutch society for physical Therapy)
Buck et al. [47]	Cross-sectional study	Anonymous electronic survey	NR	Self-reported hip or KOA	Inclusion criteria: *Patients:* (1) age ≥18 years; (2) residence in the United States; and self-reported hip or KOA *HCPs:* (1) residence in the US and (2) treated people with OA Exclusion criteria: *HCPs:* (1) no current engagement in patient care and (2) no provision of care to patients with OA	2020 to 2021	USA (CDC of the US department of health, human Services)
Horn et al. [52]	Cross-sectional study	Anonymous national online survey	NR	Self-reported diagnosis by a healthcare provider of OA of the knee, hip, or lumbar/spine	Inclusion criteria: *Patients:* (1) US residency; (2) age 18–75 years; (3) self-reported diagnosis by a healthcare provider of OA of the knee, hip, or lumbar/spine; (4) current treatment by an HCP to treat/manage OA; (5) current management of OA with lifestyle changes, over-the-counter therapies, prescription medication, or previous joint replacement surgery; (6) at least occasional joint pain and discomfort; and (7) BMI ≥30 kg/m^2^ based on self-reported height and weight. Exclusion criteria: NR	2020	USA (Novo Nordisk)
Miller et al. [37]	Qualitative study of interview data	Primary research	Single center	ICD-10 code	Inclusion criteria: *Patients*: (1) age ≥40 years; (2) a diagnosis of knee or hip OA between 2008 and 2011; and (3) treatment by the participating physician for the entire study period (2008–2015) *HCPs*: (1) practicing primary care ≥3 days per week; (2) employment at the clinic for ≥5 years; and (3) experience with treating adult patients with KOA. Exclusion criteria: NR	2008 to 2015	USA (University of Wisconsin-Madison)
Simpson 2024 [64]	RCT	Primary research	Multicenter	NR	Inclusion criteria: (1) adults aged 15–85 years; (2) on knee arthroplasty waiting list for OA with sufficient time for intervention and follow-up; (3) meet ≥1 intervention eligibility criterion: BMI ≥30 kg/m^2^, inability to perform straight leg raise/giving way, not using appropriate analgesics (unless contraindicated), or not using shock-absorbing footwear Exclusion criteria: (1) undergoing revision knee arthroplasty, fully constrained knee arthroplasty, knee replacement for a diagnosis other than osteoarthritis, or purely for pain relief (such as for those with no walking capacity); (2) had a second contralateral procedure planned within the study timeframe; (3) Were involved in another research study containing elements of behavior change related to diet, physical activity, and other study elements; (4) currently under active review with a clinician for physiotherapy; (5) unable to understand verbal explanations or written information given in English; (6) pregnant, within 4 months postpartum, or breastfeeding Additional exclusion criteria applied to included participants eligible for the weight loss aspect of the intervention included: (1) patients who had recently lost a substantial amount of weight (>5 kg in the previous 3 months); (2) already on a specialised diet; (3) patients with insulin-dependent diabetes, brittle type-2 diabetes that was being managed in secondary care (confirmed by recent glycated haemoglobin measurement if available), or moderate or severe retinopathy	2018 to 2019	UK (versus arthritis)
Tan et al. [41]	Semi-structured interviews embedded in a RCT	Secondary research	NR	NICE clinical criteria for knee OA	Inclusion criteria: (1) meet NICE clinical criteria for knee OA; (2) age ≥45 years; (3) activity-related knee pain; (4) morning knee stiffness ≤30 min or none; (5) KOOS4 ≤75; (6) kellgren–Lawrence grade >1; (7) community ambulatory with or without walking aid. Exclusion criteria: : (1) alternative diagnosis to knee OA (e.g., referred pain from spine or hip); (2) other forms of arthritis (e.g., inflammatory, post-traumatic); (3) inability to comply with protocol (e.g., cognitive impairment); (4) previous knee arthroplasty; (5) wheelchair-bound; (6) medical conditions interfering with study involvement (e.g., decompensated heart failure, stroke, end-stage renal failure).	2020	Singapore (TSH community fund, ng teng fong healthcare innovation programme)

Abbreviations: ACR = American College of Rheumatology; BASK = British Association for Surgery of the Knee; BMI = body mass index; CDC = Centers for Disease Control and Prevention; EMR = electronic medical record; ERDF = European Regional Development Fund; EULAR = European Alliance of Associations for Rheumatology; GP = general practitioner; HCP = healthcare professional; ICD-10-CM = ICD-10-Clinical Modification; ICPC-2-R = International Classification of Primary Care, 2nd ed rev.; KOA = osteoarthritis of the knee; NICE = National Institute for Health and Care Excellence; NR = not reported; NRS = numerical rating scale; OA = osteoarthritis; PT = physical therapist; RCT = randomized controlled trial; TKA = total knee arthroplasty.

### 3.3 Patient and HCP characteristics

Across the studies investigating patients' perceptions, patient populations were predominantly overweight or obese adults with variable KOA severity. Pain and function were commonly assessed using the Western Ontario and McMaster Universities Arthritis Index (WOMAC), Knee injury and Osteoarthritis Outcome Score, numerical rating scales and visual analogue scales. In studies reporting the patients’ race or ethnicity, more than half (4/7 studies) were conducted in a predominantly White (≥60% of total patients) population [47,52,65,67]. When reported, disease duration ranged from <6 months to >15 years [26,27,32,34,40,41,44,49,54,56,58,60,61].

Studies that reported HCPs’ perceptions recruited general practitioners (GPs) and physicians (n = 4) [28,37,45,51], physical therapists (PTs) (n = 6) [23,24,29,36,38,55], a mixture of HCPs (n = 2) [41,44], surgeons (n = 2) [30,51], dietitians (n = 1) [35], nurses (n = 1) or both dietitians and physiotherapists (n = 1) [32]. The full details of the patient and HCP characteristics are provided in Supplementary Tables 2 and 3, respectively.

### 3.4 Quality assessment

The Critical Appraisal Skills Programme tool was used to assess 23 qualitative studies [[22], [23], [24], [25], [26], [27], [28], [29], [30], [31], [32], [33], [34], [35], [36], [37], [38], [39], [40], [41], [42], [43], [44]], while the Newcastle-Ottawa scale was used to assess 15 cross-sectional [[45], [46], [47], [48], [49], [50], [51], [52], [53], [54], [55], [56], [57], [58], [59]], one quasi-experimental and one observational cohort studies [66,67], and the Cochrane risk-of-bias tool 2.0 was used to assess six RCTs [[60], [61], [62], [63], [64], [65]]. All of the qualitative studies scored positively in nine of the 10 domains and were considered of good quality. Eleven of the 18 studies assessed using the NOS scored poorly [44,[46], [47], [48], [49], [50], [51], [52], [53], [54],^56^], likely due to the semi-quantitative nature of these studies, while the other seven studies had a satisfactory score [41,45,55,[57], [58], [59],^67^]. Three of the studies assessed using the RoB 2.0 were of low risk of bias [60,61,64] while two studies had some concerns [62,65], and another study was at high risk of bias (Supplementary Tables 4–5) [63].

### 3.5 Patient-reported barriers to achieving weight reduction

**Fig. 2:**
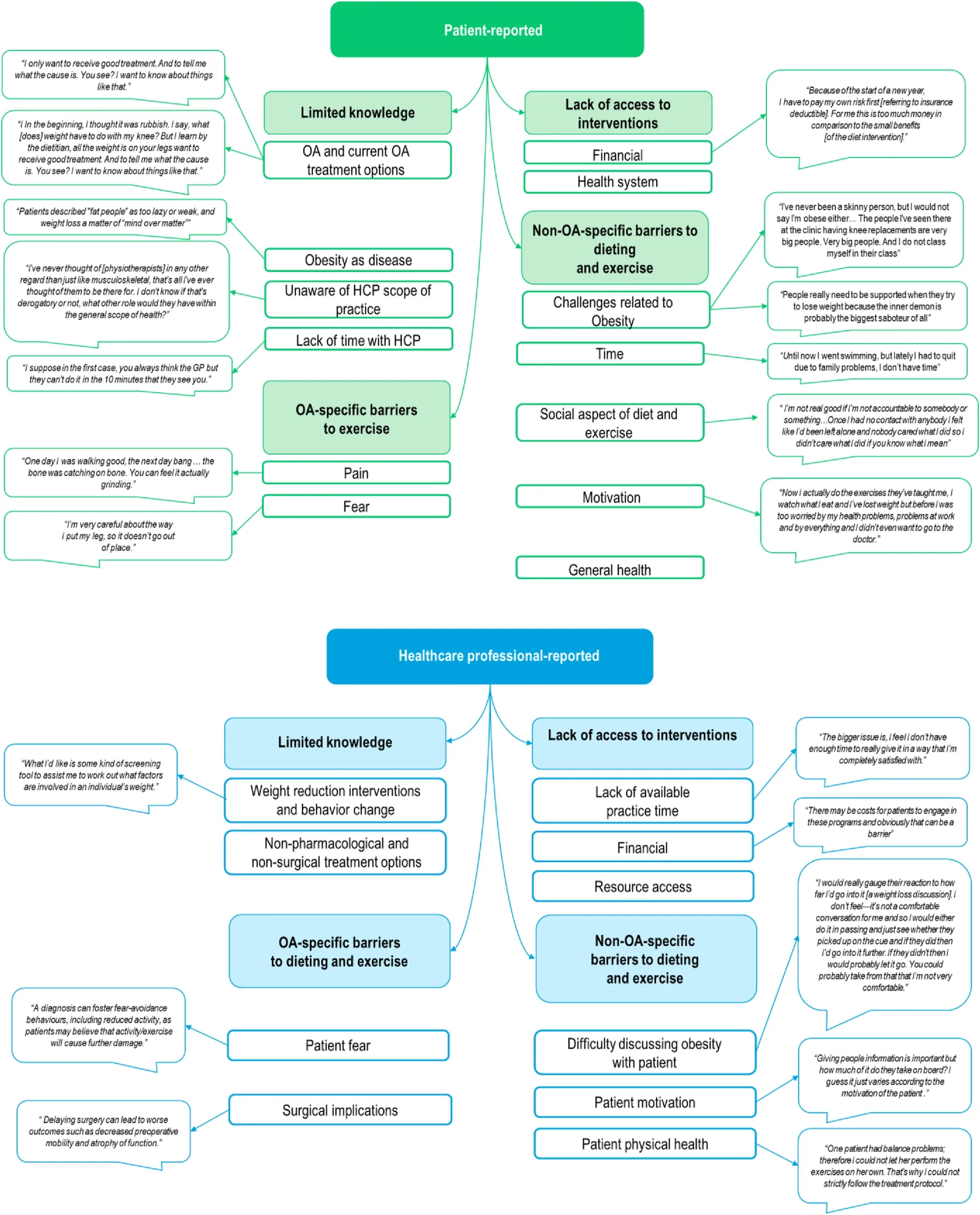
Patient- and HCP-reported Barriers to Weight Reduction in Knee Osteoarthritis. Abbreviations: HCP = healthcare professionals; OA = osteoarthritis.

The following themes related to barriers to weight reduction were identified across the studies: limited knowledge, lack of access to interventions, OA-specific barriers to exercise, and non-OA-specific barriers to dieting and exercise in patients with KOA (Fig. 2).

#### 3.5.1 Limited knowledge

Three studies reported that patients considered insufficient education on OA and OA treatments to be a barrier to weight reduction. Patients reported that a lack of educational resources [37] and inadequate detail in dietary advice [27] were barriers to weight reduction. Furthermore, patients reported receiving conflicting information on weight reduction for OA treatment from medical providers [37], exacerbating limited knowledge as a barrier to weight reduction.

Patients with OA and obesity did not always view themselves to have obesity [39,52], which may be a barrier to weight reduction as a mechanism to reduce KOA symptoms [39]. Patients’ lack of knowledge regarding the role of specific HCPs (i.e., physiotherapists) can serve as a potential barrier [22]. Several patients did not consider physiotherapists knowledgeable about weight reduction because they were unaware of their scope of practice, knowledge, and skill set [22]. The studies did not discuss obesity specialists or provide detailed information regarding pharmacological and surgical approaches for weight reduction.

Patients' lack of knowledge may be partially attributable to inadequate time spent with HCPs [22], often resulting in patients having unanswered questions regarding OA treatment [35,37]. However, information regarding patients’ questions specifically related to obesity management and treatment was not provided.

#### 3.5.2 Lack of access to interventions

Lack of access to interventions was reported by patients as a barrier to weight reduction in terms of resources, costs, and support [22,27,31,46,69]. Barriers in healthcare services included long waiting times, poor access to treatment (e.g. dietary advice) and lack of referral to appropriate HCPs (e.g., dietitians) [27]. Impeded costs reported by patients included treatment (e.g., exercise classes, gym memberships) [32,37,48] and insurance concerns (e.g., patient contribution costs or limitations imposed by insurance providers) [27,32]. Finally, lack of access to interventions may further hinder long-term weight reduction, since patients reported experiencing heightened anxiety when faced with the prospect of weight self-management without the support of an HCP [35].

#### 3.5.3 KOA-specific barriers to exercise

Patients reported that pain was a barrier to engaging in physical activity [25,26,37,43,53,66]. Knee pain may prevent exercise, which may contribute to increased body weight, and both directly and indirectly increase knee pain [25]. Some patients reported that pain in other joints prevented them from undertaking physical activity [33,53]. Fear was another barrier to exercise: patients reported that they were afraid of falling or further joint damage [25,48,66].

#### 3.5.4 Non-KOA-specific barriers to dieting and exercise

The following themes were identified among the non-OA-specific barriers: intrapersonal, physical, and social. The patient-reported intrapersonal barriers included difficulty achieving weight reduction, lack of results, motivation, time, and forgetting to exercise. The difficulty of achieving weight reduction was a barrier [22,26,37,40,43,46] with patients believing that weight reduction was impossible [26], a waste of time [43], or something they could not do [46]. Patients discontinued dieting because of a lack of short- and long-term results [31], problems with calorie counting, eating out of boredom, and initial unpleasant side effects of a new diet [35,43,66]. A lack of motivation and readiness to lose weight was noted as a barrier to dieting and exercise [32,33,[39], [40], [41],^53^,^64^]. People felt that to successfully lose and maintain weight, there was a need for accountability. Where individuals had participated in weight loss interventions, they found that once they had finished the program they returned to old habits [33]. Patients considered private weight-reduction centers misleading because of the high costs and failure to meet expectations, further negatively impacting their motivation to engage in future weight-reduction attempts [31]. A lack of time was reported as a barrier to dieting and/or exercise [32,48,53,66]. Patients lacked time because of work [66] or caring for others [48], resulting in failure to adhere to exercise programs, dietary advice, or HCP appointments [32]. Additional barriers to exercise included forgetting [66], lack of desire to engage in any additional new physical activity [48], or lack of self-perception as the sporty type (16%) [48].

Patient-reported physical barriers included their general health, age, and challenges with mobility. Patients listed their general health and tiredness as barriers to dieting and exercise [33,37,43,48,52,66]. Patients believed that they were too old to exercise or that an increased sedentary lifestyle was a normal part of aging [43,48]. Challenges with general movement, including difficulty walking [66] and limited mobility [37], prevented some patients from exercising. Disrupted sleep and mood changes were also listed as barriers [37] that contribute to the lack of energy [48] and negative emotions [26] which impair engagement in adequate physical activity.

Patient-reported social barriers included the social aspect of eating and insufficient social support. Patients reported that adhering to dietary advice was hindered by their family's eating habits, preparing meals with others, social situations (e.g., holidays) [33,35,66], and relationship changes [37]. Additionally, patients highlighted insufficient social support as a barrier to exercise participation [66] and that they would feel more motivated if they could exercise in a group [26].

### 3.6 HCP-reported barriers to achieving weight reduction

#### 3.6.1 Limited knowledge

HCPs (GPs, PTs), reported they had insufficient knowledge regarding weight-reduction interventions and facilitating behavior change [24,28,36,37], resulting in HCPs recommending simple solutions that are not aligned with current guidelines [56] or passing on incorrect perceptions of OA treatment (including dietary advice) and disease progression to patients with KOA [37]. Even within planned research programmes, research nurses and PTs highlighted the need for further training for them to feel properly equipped to deliver the weight loss interventions [23,64]. Research nurses in particular, expressed the need for more in-depth understanding or support with the psychological/behavior-change techniques to deliver the intervention more effectively [64]. HCPs showed a willingness to improve their knowledge via tailored education programs [28]. HCPs reported that a lack of patient knowledge of OA management was a barrier to weight reduction and that patient education is essential for self-management [28,37,38]. HCPs often provided patients with simplistic, biomedical descriptions of OA that overlooked broader psychosocial factors [28]. Patients may receive incorrect information from other sources (e.g., friends, family, and the media), which lead to ideas that are misaligned with HCP recommendations [28], making it difficult to convince patients of the value of nonpharmacological treatment options (e.g., diet and exercise) [37].

#### 3.6.2 Lack of access to interventions

HCPs reported inadequate time to provide effective weight-reduction management [28,32,37], including session duration [37] and number of sessions [32]. Consequently, HCPs lacked the time to provide personalized treatment options and address patient issues [28,37]. HCPs considered financial factors (e.g., the cost of weight-reduction programs, insurance coverage, and travel expenses) to be barriers to patient weight reduction [28,37].

HCPs further reported that access to weight-reduction resources, such as weight-reduction programs, educational resources, and appointments with HCPs, were barriers to weight reduction [28,37,64]. These problems were likely exacerbated by poor availability in remote locations and long waiting times [28]. In contrast, some HCPs believed that sufficient resources were available but that patients were unaware of them [28].

#### 3.6.3 OA-specific barriers to dieting and exercise

Some HCPs believed that an OA diagnosis can lead to fear-avoidance behaviors, resulting in reduced physical activity to avoid further damage [28]. There was also concern that some weight loss initiatives may create problems that would complicate KOA surgery or affect the health of patients [64] and may increase potential risks to the patient if surgery is delayed [30].

#### 3.6.4 Non-OA-specific barriers to dieting and exercise

HCPs often reported the difficulty of raising the topic of weight reduction [24,28,32,36] primarily because they were concerned with negatively influencing their relationship with the patient [28,32] and lacked the skills to address this topic appropriately [32]. Consequently, some HCPs avoided discussing weight reduction altogether [28]. HCPs identified low patient motivation as a key barrier to patient weight reduction [28,32] which could result in a reciprocal loss of their motivation and their reluctance to recommend these types of interventions [28]. Patients’ physical health, including comorbidities that may influence treatment and adherence to weight-reduction programs [32] and inability to participate in exercise or access HCP services [37], were also considered barriers to weight reduction [32,37].

### 3.7 Patient and HCP expectations of the impact of weight reduction on OA outcomes

Patients believed that weight reduction was effective for improving KOA symptoms [25,37,44]. Most patients stated that incorporating dietary changes and weight-reduction programs would alleviate stiffness and pain [37], which would improve their KOA symptoms by reducing the load exerted on the joint [25]. In one RCT where patients were receiving dietary advice through video calls and a mobile application, patients reported that they would expect the weight loss to result in improved eligibility for surgery [63]. HCPs exhibited a range of inconsistent views, expressing both skepticism (some GPs did not see improvements in patients with OA following exercise and weight management advice) [28] and support [36,44] for the effectiveness of weight reduction as a therapeutic approach for KOA.

### 3.8 Patient and HCP expectations of weight reduction delivery

Patients expected HCPs to have sufficient experience in weight-reduction counseling, have a good rapport with patients, and deliver a holistic, patient-centered weight-reduction intervention [22,34]. Patients expected interventions to incorporate a multidisciplinary team including PTs/physiotherapists and GPs [22,24] and involve specific interventions (e.g., exercise) [54]. Patients felt a responsibility to actively participate in their weight reduction management [52].

Most HCPs believed that weight reduction should be a first-line treatment for KOA and that weight-reduction interventions should involve a multidisciplinary team [41,50,51]. Most HCPs also expressed that they should actively contribute to educating [38] and supporting patients’ weight management [52] and emphasized the importance of personalized, evidence-based interventions with a high level of supervision by an experienced HCP [51]. Some HCPs also suggested a need to transition from multidisciplinary to a transdisciplinary approach where a single person with multiple skill sets would deliver the intervention independently [41]; however, other HCPs were concerned about their ability to achieve this [64].

### 3.9 Patient and HCP satisfaction with weight reduction interventions

Data regarding patient and HCP satisfaction with weight reduction interventions were scarce. Most patients expressed satisfaction with the level of care they received both within [64] and outside of the clinical trial setting [34,45], as well as satisfaction with the weight loss and the motivation which ensues from the achieved weight loss [35]. Patients perceived primary care physicians as the most helpful HCPs relative to other specialties [52].

HCPs indicated that dietary classes helped empower patients by improving their dietary knowledge, highlighting the benefits of weight loss for improving pain and function and giving patients theunc skill sets for self-management [41].

## 4 Discussion

Although previous literature reviews have reported on the barriers of lifestyle changes for weight reduction in patients with KOA, it is important to also consider barriers and perceptions for the surgical and pharmacological treatment of weight reduction in patients with KOA. This literature review was conducted to gain a better understanding of patient and HCP perceptions of and barriers to surgical, pharmacological and non-pharmacological weight reduction approaches. This is an important area to address, given that weight reduction is a core recommended treatment for people with KOA. To our knowledge, this is the first systematic review to synthesize perceptions of weight loss for surgical, pharmacological and non-pharmacological approaches in this population.

Patients and HCPs shared many common perceptions, including holistic, patient-centered interventions involving a multidisciplinary team.

Enhancing patient knowledge through improved access to educational resources, increasing time with HCPs, and bolstering HCP knowledge through appropriate training are essential steps that can contribute to more patients with KOA achieving weight reduction, a significant barrier expressed by both patients and HCPs [27,28,37,56]. Patients specifically highlighted the scarcity of information regarding weight-reduction interventions [37], which could be partially attributed to limited access to educational materials [37], insufficient time with HCPs, or unawareness of HCP specialties’ roles in achieving weight reduction [22]. HCPs acknowledged that inadequate patient knowledge can hinder weight reduction efforts, including information from unreliable sources that contradicts HCP recommendations [28]. Furthermore, some HCPs lacked adequate knowledge of weight-reduction interventions for KOA, resulting in their discomfort in delivering these interventions [24,28,36,37].

The HCP-perceived barriers identified were generally consistent with previously published HCP-reported barriers to lifestyle intervention implementation in patients with KOA [13,70]. The barriers reported in the literature include individual, interpersonal, and societal factors [70]. In particular, patients' reluctance to engage in lifestyle interventions and their limited health literacy, and HCPs’ limited knowledge of and skills to facilitate lifestyle modification, were among the key barriers [13]. Nevertheless, we have identified an important gap in the synthesis of evidence for both pharmacological and surgical approaches for which perceptions and barriers specific to patients with KOA were not identified.

The use of obesity management medications (OMMs) was not raised as a treatment option in the included studies, which were performed between 2004 and 2025, before the use of modern OMM for weight management. Compared with lifestyle modification, which is associated with a weight reduction of approximately 5%–10%, greater weight reduction can be achieved with OMMs. For example, semaglutide (a glucagon-like peptide 1 receptor agonist) and tirzepatide (a dual glucose-dependent insulinotropic polypeptide/glucagon-like peptide 1 receptor agonist) are associated with weight reductions of approximately 15% and 20.9%, respectively [71]. However, there are some safety concerns in the use of OMMs for treating obesity, such as an increased risk of sarcopenia, especially in older adults [72]. In addition, concerns regarding access and reimbursement are barriers to the use of OMMs for the treatment of obesity and diabetes with discordant approaches seen across healthcare systems. [73]. Furthermore, OMMs have not been reported as a cost-effective option compared to no intervention from a healthcare/payer perspective for treating obesity in patients without diabetes in high-income countries [74]. In the context of OA, OMMs are not approved for use for the treatment of OA, and clinical trial data in patients with OA are limited [75]. In the recent phase 3 STEP 9 study (NCT05064735), semaglutide alleviated knee pain in patients with KOA and obesity [76]. Further research and clinical trials are essential for evaluating the efficacy of OMMs in treating KOA, which could potentially lead to their approval for this indication and their incorporation into evidence-based international guidelines. In this regard, TRIUMPH-4 is an ongoing trial aiming to evaluate the efficacy and safety of retatrutide in participants with obesity or are overweight and have KOA [77].

Other than the inherent limitations of SLRs (e.g., heterogeneity, publication bias, selection bias), there were also several limitations of the current body of literature investigating patient and HCP perceptions of weight reduction. First, several publications did not consider KOA independently of other conditions (e.g. hip OA) and were not included in this SLR despite addressing areas of interest [[78], [79], [80]]. Second, most studies included a predominantly White population with a sample size of fewer than 50 patients. The barriers faced by patients from other ethnic and cultural backgrounds may differ from those identified in the current SLR, and the identified barriers may not be representative of the wider KOA population. Third, the included studies, which focused on patients with KOA rather than weight reduction in general, did not address the role of obesity specialists or pharmacological/surgical interventions. By neglecting these broader aspects, the studies revealed a significant gap in adopting a holistic approach to patient management. Finally, not all included studies explicitly mentioned “weight reduction” as the primary treatment goal, but it can be inferred that dietary interventions and exercise were undertaken to achieve weight reduction or as a part of KOA treatment.

## 5 Conclusion

Patients and HCPs agreed that weight reduction is effective for improving KOA and identified substantial knowledge, behavioral and healthcare system barriers. This SLR highlights the need for open discussions between patients and HCPs, training for HCPs, and educating patients that it is safe to be active and exercise within the limits of their joint pain. Improving patient and HCP knowledge through appropriate training is also crucial to ensure that more patients with KOA receive consideration for weight-reduction treatments. This study highlights the unmet need for potential interventions that can assist patients and clinicians in meeting weight reduction goals in patients with KOA.

## Author contributions

PGC designed the study and made critical revision of the manuscript for important intellectual content; CR designed the study, conducted the research, drafted the manuscript, and made critical revision of the manuscript for important intellectual content; JF conducted the research and made critical revision of the manuscript for important intellectual content; LL designed the study, conducted the research, and made critical revision of the manuscript for important intellectual content; KG designed the study and made critical revision of the manuscript for important intellectual content; RR designed the study and made critical revision of the manuscript for important intellectual content; MPN designed the study and made critical revision of the manuscript for important intellectual content. All authors have read and approved the final version of the manuscript.

## Funding

This study was sponsored by Eli Lilly.

## Declaration of competing interest

PGC has done consultancies or speakers bureaus for AbbVie, Eli Lilly, Formation Bio, Galapagos, Genascence, GlaxoSmithKline, Grünenthal, Janssen, Levicept, Novartis, Pacira, Stryker, and Takeda.

Generative AI and AI-assisted technologies were NOT used in the preparation of this work.
