# Supplementary material for: Patient and healthcare professional perceptions of weight reduction in the treatment of knee osteoarthritis: A systematic literature review
Source: Osteoarthr Cartil Open. 2026 Sep 7;8(4):100875. doi: 10.1016/j.ocarto.2026.100875 (PMC13589083; doi:10.1016/j.ocarto.2026.100875)
Supplement: Supplementary file 1 [file mmc1.docx]

**Supplementary Table 1**. Search Strategies

| **Search Number** | **Search Terms** | **Results** |
| --- | --- | --- |
| **Search Strategy for Embase** | |  |
| 1 | exp osteoarthritis/ or (osteoarthriti$ or osteo-arthritis or degenerative arthritis or degenerative joint).ti,ab. or OA.ti. | 224255 |
| 2 | body weight loss/ | 282690 |
| 3 | ((weight or pound$ or kilogram$ or stone$ or body mass) adj2 (decrease$ or reduc$ or losing or loss or watch$)).ti,ab. | 283231 |
| 4 | 2 or 3 | 383265 |
| 5 | 1 and 4 | 4131 |
| 6 | 5 not ((exp animal/ or nonhuman/) not exp human/) | 3878 |
| 7 | (case report or case series or woman or man or child or adolescent or female or male or boy or girl or infant).ti. or exp case report/ or exp case study/ or case report$.jn. or case report$.jx. | 3877731 |
| 8 | (Ephemera or "Introductory Journal Article" or News or "Newspaper Article" or Editorial or Comment or Overall).pt. or in vitro Techniques/ or in vitro study/ or (commentary or editorial or comment or letter or mice or rat or mouse or animal or murine).ti. | 4072062 |
| 9 | review.pt. not (systematic or (meta and analy*) or ((indirect or mixed) and 'treatment comparison')).ti,ab. | 3135870 |
| 10 | conference abstract.pt. | 5703332 |
| 11 | clinical trials repository.su. | 552954 |
| 12 | or/7-10 | 16079994 |
| 13 | 6 not 11 | 1648 |
| 13 | limit 12 to yr="2010 -Current" | 1231 |
| **Search Strategy for Medline** | |  |
| 1 | exp osteoarthritis/ or (osteoarthriti$ or osteo-arthritis or degenerative arthritis or degenerative joint).ti,ab. or OA.ti. | 133503 |
| 2 | weight loss/ or weight reduction programs/ | 49978 |
| 3 | ((weight or pound$ or kilogram$ or stone$ or body mass) adj2 (decrease$ or reduc$ or losing or loss or watch$)).ti,ab. | 174871 |
| 4 | 2 or 3 | 186078 |
| 5 | 1 and 4 | 1376 |
| 6 | 5 not (exp animals/ not exp humans/) | 1293 |
| 7 | (case report or case series or woman or man or child or adolescent or female or male or boy or girl or infant).ti. or exp case reports/ or case report$.jn. or case report$.jw. | 3152403 |
| 8 | (Ephemera or "Introductory Journal Article" or News or "Newspaper Article" or Editorial or Comment or Overall).pt. or in vitro Techniques/ or in vitro study/ or (commentary or editorial or comment or letter or mice or rat or mouse or animal or murine).ti. | 3572105 |
| 9 | review.pt. not (systematic or (meta and analy*) or ((indirect or mixed) and 'treatment comparison')).ti,ab. | 3273043 |
| 10 | or/7-9 | 9665794 |
| 11 | 6 not 10 | 887 |
| 12 | limit 11 to yr="2010 -Current" | 162 |
| **Search Strategy for the CENTRAL Register of Controlled Trials** | |  |
| 1 | exp osteoarthritis/ or (osteoarthriti$ or osteo-arthritis or degenerative arthritis or degenerative joint).ti,ab. or OA.ti. | 26041 |
| 2 | weight loss/ or weight reduction programs/ | 9442 |
| 3 | ((weight or pound$ or kilogram$ or stone$ or body mass) adj2 (decrease$ or reduc$ or losing or loss or watch$)).ti,ab. | 31768 |
| 4 | 2 or 3 | 32965 |
| 5 | 1 and 4 | 501 |
| 6 | (EUCTR$ or NCT$ or ICTRP$ or CTRI$ or ISRCTN$ or chictr$ or actrn$ or IRCT$ or NTR$).tn. | 655204 |
| 7 | (clinical trial protocol or conference proceeding or dissertation thesis or trial registry record or trial registry record clinical trial protocol).pt. | 936428 |
| 8 | 5 not (6 or 7) | 131 |
| 9 | limit 8 to yr="2010 -Current" | 103 |
| **Search Strategy for the Cochrane Database of Systematic Reviews** | |  |
| 1 | (osteoarthriti$ or osteo-arthritis or degenerative arthritis or degenerative joint).ti,ab. or OA.ti. | 124 |
| 2 | ((weight or pound$ or kilogram$ or stone$ or body mass) adj2 (decrease$ or reduc$ or losing or loss or watch$)).ti,ab. | 159 |
| 3 | 1 and 2 | 3 |
| 4 | limit 3 to yr="2010-current" | 3 |
| **Search Strategy for the APA PsycINFO** | |  |
| 1 | exp osteoarthritis/ or (osteoarthriti$ or osteo-arthritis or degenerative arthritis or degenerative joint).ti,ab. or OA.ti. | 2651 |
| 2 | weight loss/ or weight reduction programs/ | 5171 |
| 3 | ((weight or pound$ or kilogram$ or stone$ or body mass) adj2 (decrease$ or reduc$ or losing or loss or watch$)).ti,ab. | 18110 |
| 4 | 2 or 3 | 18396 |
| 5 | 1 and 4 | 62 |
| 6 | dissertation abstract.pt. | 600715 |
| 7 | 5 not 6 | 50 |
| 8 | limit 7 to yr="2010-current" | 35 |

Supplementary Table 2. Summary of Patient Characteristics from Included StudiesD2

| **Author, Year** | **Study Arm/ Sample Size** | **Age (years)** | **Male, n (%)** | **BMI (kg/m^2^)** | **Race/Ethnicity, n (%)** | **KOA Severity** | **Pain Severity** | **Functional Severity** | **Disease Duration (years)** |
| --- | --- | --- | --- | --- | --- | --- | --- | --- | --- |
| **Australia** | | | | | | | | | |
| Allison et al. [17] | Entire cohort  n=13 | Mean (SD): 60.5 (8.3) | 5 (39) | Mean (SD): 34.3 (6.1) | NR | NR | NR | NR | NR |
| Allison, 2024 [55] | Very low energy plus exercise intervention n=42 | Mean (SD): 60.5 (7.0) | 15 (36) | 35.2 (5.3) | NR | NR | 9.1 (3.5) | 23.1 (10.9) | Knee symptoms duration: 3.0 (1.0–8.0) |
|  | Exercise only control  n=46 | Mean (SD): 60.0 (8.3) | 17 (37) | 35.3 (5.3) | NR | NR | 8.7 (4.6) | 26.5 (11.4) | Knee symptoms duration: 3.0 (1.0–10.0) |
| Bennell et al. [56] | Control  n=67 | Mean (SD): 65.3 (8.7) | 22 (32.8) | Mean (SD): 33.8 (3.6) | NR | NR | Average overall knee pain (NRS), mean (SD): 5.9 (1.3) | WOMAC knee symptoms severity, mean (SD) –  Function: 22.7 (10.2) | Median symptom duration (IQR), years: 4 (2-10) |
|  | Exercise  n=172 | Mean (SD): 65.4 (8.2) | 79 (45.9) | Mean (SD): 33.1 (3.6) | NR | NR | Average overall knee pain (NRS), mean (SD): 5.9 (1.3) | WOMAC knee symptoms severity, mean (SD) –  Function: 22.8 (10.6) | Median symptom duration (IQR), years: 5 (2-10) |
|  | Diet and Exercise  n=175 | Mean (SD): 64.1 (8.1) | 86 (49.1) | Mean (SD): 33.0 (3.3) | NR | NR | Average overall knee pain (NRS), mean (SD): 5.9 (1.4) | WOMAC knee symptoms severity, mean (SD) –  Function: 24.0 (8.9 | Median symptom duration (IQR), years: 5 (3-10) |
| Bopf et al. [41] | Entire cohort  n=95 | Mean (SD): 64.45 (8.61) | 36 (38) | NR | NR | NR | NR | NR | NR |
| Bunzli et al. [20] | Entire cohort  n=27 | Mean (SD): 68 (NR) | 14 (52) | Mean (SD): 33 (NR) | NR | NR | NR | NR | NR |
| Ekram et al. [43] | Normal weight  n=17 | Mean (SD): 71.0 (11.0) | 5 (19.4) | Mean (SD): 23.0 (2.2) | NR | NR | WOMAC knee symptoms severity, mean (SD) –  Pain: 29.1 (11.5)  Stiffness: 11.6 (5.9)  Function: 102.4 (35.0) | NR | NR |
|  | Overweight  n=17 | Mean (SD): 67.5 (12.2) | 14 (50) | Mean (SD): 27.8 (1.5) | NR | NR | WOMAC knee symptoms severity, mean (SD) –  Pain: 28.1 (10.4)  Stiffness: 11.3 (5.8)  Function: 92.9 (39.4) | NR | NR |
|  | Obese  n=17 | Mean (SD): 65.1 (11.1) | 14 (24.6) | Mean (SD): 39.2 (9.0) | NR | NR | WOMAC knee symptoms severity, mean (SD) –  Pain: 26.7 (16.2)  Stiffness: 10.7 (5.4)  Function: 91.5 (41.0) | NR | NR |
| Lawford et al. [30] | Entire cohort  n=24 | Mean (SD): 63 (9) | 11 (46) | Mean (SD): 32.6 (2.9) | NR | NR | Knee pain measured by 11-point numeric rating scale, mean (SD): 6.2 (1.4) | WOMAC physical function, mean (SD): 26.8 (7.7) | NR |
| Lawford et al. [28] | Entire cohort n=20 | Mean (SD): 65 (9) | 10 (50) | 32.7 (2.9) | NR | NR | Measured by 11-point numeric rating scale (0=no pain, 10=worst pain possible): 5.6 (1.4) | WOMAC PF: 22 (5.1) | NR |
| Lawford, 2024 [29] | Entire cohort n=42 | Mean (SD): 60.5 (7.0) | 15 (36) | 35.2 (5.3) | NR | NR | Knee pain during walking over the past week ≥4 on an 11-point numeric rating scale: 22 (100%) | NR | Median 3.0 (IQR 1.0-8.0) years |
| Pavlovic et al. [34] | Entire cohort n=25 | NR | 7 (28) | NR | NR | End-stage osteoarthritis | NR | NR | NR |
| Wallis et al. [39] | Entire cohort  n=53 | NR | 15 (28) | NR | NR | NR | NR | NR | n (%) –  <5 years: 20 (38)  Between 5 and 10 years: 19 (36)  Between 11 and 15 years: 8 (15)  16 or more years: 6 (11) |
| **Denmark** | | | | | | | | | |
| Baumbach et al. [40] | Entire cohort  n=131 | Mean (95% CI): 63 (40-85) | 61 (47) | NR | NR | NR | NR | Knee related quality of life score, mean (95% CI): 48 (13-87) | NR |
| Ingelsrud et al. [49] | Entire cohort  n=517 | Mean (SD): 67 (11) | 193 (37) | Mean (SD): 30 (5) | NR | OKS mean (SD): 23 (8), for n=501  KL grade (n=459), n (%)  0: 1 (0)  1: 29 (6)  2: 89 (19)  3: 171 (37)  4: 169 (37) | Knee pain (VAS) median (IQR): 7 (5–8) | NR | KOA duration (n=512), n (%)  0-6 months: 43 (8)  6-12 months: 49 (10)  1-2 years: 73 (14)  2-5 years: 105 (21)  5-10 years: 122 (24)  >10 years: 120 (23) |
| **France** | | | | | | | | | |
| Richette et al. [53] | All patients  n=1821 | Mean (SD): 67.3 (9.7) | 798 (43.8) | Mean (SD): 28.9 (4.7) | NR | NR | Pain (VAS), mean (SD) –  On movement over the last 24 hr: 61.6 (17.9)  At rest: 32.7 (20.0)  Global pain: 49.3 (16.4)  Pain at night: 47.1% | NR | Mean (SD): 7.7 (5.7) |
|  | GP patients  n=1570 | Mean (SD): 67.0 (9.7) | 703 (44.8) | Mean (SD): 29.1 (4.6) | NR | NR | Pain (VAS), mean (SD) –  On movement over the last 24 hr: 62.4 (17.6)  At rest: 33.9 (20.5)  Global pain: 49.8 (16.3)  Pain at night: 49.9% | NR | Mean (SD): 7.8 (5.6) |
|  | Rheumatologist patients  n=251 | Mean (SD): 69.8 (9.7) | 95 (37.8) | Mean (SD): 28.0 (4.9) | NR | NR | Pain (VAS), mean (SD) –  On movement over the last 24 hr: 57.3 (19.0)  At rest: 25.3 (20.6)  Global pain: 46.2 (17.1)  Pain at night: 29.7 | NR | Mean (SD): 6.8 (5.4) |
| **India** | | | | | | | | | |
| Siddique, 2025 [54] | Diet and weight reduction strategy arm n=39 | Mean (SD): 58.5 (7.7) | 0 (0) | NR | Asian: 39 (100) | NR | Mean WOMAC pain score (SD NR): 6 | Mean WOMAC function score (SD NR): 18.9 | NR |
| **International** | | | | | | | | | |
| Østerås et al. [52] | Entire cohort  n=354 | Mean (SD): 67.7 (10.07) | 117 (33) | Mean (SD): 28.5 (4.78) | NR | NR | WOMAC SF-8, mean (SD): 40.4 (20.65) | NR | NR |
|  | Norway  n=71 | Mean (SD): 67.7 (10.07) | 23 (47) | Mean (SD): 28.4 (4.22) | NR | NR | WOMAC SF-8, mean (SD): 46.4 (19.17) | NR | NR |
|  | Denmark  n=49 | Mean (SD): 57.6 (7.55) | 23 (47) | Mean (SD): 27.3 (2.58) | NR | NR | WOMAC SF-8, mean (SD): 31.6 (16.65) | NR | NR |
|  | Portugal  n=135 | Mean (SD): 69.2 (8.15) | 31 (23) | Mean (SD): 29.5 (5.23) | NR | NR | WOMAC SF-8, mean (SD): 28.7 (22.53) | NR | NR |
|  | UK  n=99 | Mean (SD): 71.7 (10.07) | 40 (40) | Mean (SD): 28.0 (5.20) | NR | NR | WOMAC SF-8, mean (SD): 45.8 (19.58) | NR | NR |
| **The Netherlands** | | | | | | | | | |
| Knoop et al. [27] | Entire cohort  n=15 | Mean (SD): 62 (11) | 2 (13) | NR | NR | NR | Numeric rating scale, mean (SD): 5 (2) | NR | Mean (SD): 13 (12) |
| Oomen et al. [51] | Entire cohort  n=434 | Mean (SD): 63.3 (8.8) | 129 (30) | Mean (SD): 27.8 (4.8) | NR | NR | KOOS – daily functioning, mean (SD): 63.5 (18)  Pain VAS, mean (SD): 5.4 (2.1) | NR | Duration of symptoms, n (%) –  Less than one year: 30 (7)  Between one and five years: 163 (38)  More than five years: 241 (56) |
| **Norway** | | | | | | | | | |
| Grønhaug et al. [44] | Entire cohort  n=71 | Mean (SD): 66 (10) | 23 (32) | Mean (SD): 28 (5.3) | NR | NR | HOOS/KOOS subscores, n (%) –  Pain: 52 (17)  Symptoms: 60 (15)  Function in daily living: 52 (22)  Function in sport and recreation: 21 (20)  Hip/knee-related quality of life: 36 (19) | NR | Time since OA diagnosis, n (%) –  <1 year: 12 (17)  1-3 years: 16 (23)  4-6 years: 17 (24)  7-10 years: 11 (15)  >10 years: 14 (20)  Missing: 1 (1) |
| **Singapore** |  |  |  |  |  |  |  |  |  |
| Tan, 2025 [36] | Dietetics and Nutrition (NND) component of the CONNACT intervention arm: n=22 | Mean (SD): 68.3 (6.92) | 8 (36.4) | NR | NR | Kellgren-Lawrence Score >1: 22 (100) | NR | NR | NR |
| Tan, 2025 [37] | Entire cohort: n=314 | Mean (SD): 63.38 (8.13) | 105 (33.4) | 26.05 (5.98) (n=306) | NR | NR | KOOS-12 Pain score, mean (SD): 63.82 (17.84) | KOOS-12 Function in daily living (ADL) score, mean (SD): 74.14 (19.37) | Time since OA diagnosis (n=313), Mean (SD)  < 1 year: 45 (14.4)  1 to 3 years: 103 (32.9)  4 to 6 years: 70 (22.4)  7–10 years: 69 (22.0)  > 10 years: 26 (8.3) |
| **South Africa** | | | | | | | | | |
| Coetzee et al. [22] | Entire cohort  n=16 | Median (range): 67.5 (49-84) | 1 (6.25) | NR | NR | NR | NR | NR | Years living with OA, n (%) –  1-5: 3 (18.75)  6-10: 2 (12.5)  11-15: 2 (12.5)  16-20: 0 (0)  >20: 9 (56.25) |
| **Spain** | | | | | | | | | |
| Carmona-Teres et al. [21] | Entire cohort  n=10 | Mean (SD): 71 (10) | 3 (30) | NR | NR | NR | NR | NR | 11 (7) |
| Isla Pera et al. [26] | Entire cohort  n=10 | Mean (SD): 67.23 (7.87) | 2 (20) | Mean (SD): 40.47 (4.22) | NR | NR | NR | NR | NR |
| **Thailand** | | | | | | | | | |
| Aree-Ue et al. [61] | Intervention group (MUFIP)  n=38 | Mean (SD): 67.08 (6.73) | NR | NR | NR | NR | Pain (Visual Analog Scale), mean (SD)  Left knee pain: 5.9 (2.1)  Right knee pain: 6.3 (0.9) | TUG score, mean (SD):  12.7 (1.9) | NR |
| **United Kingdom** | | | | | | | | | |
| Howarth et al. [48] | Entire cohort  n=45 | Mean (range): 67 (50–87) | 20 (44) | Mean (range): 34 (30–45) | NR | NR | NR | NR | NR |
| Simpson, 2024 [59] | Weight loss component of package-of-care intervention: n=28 | NR | NR | ≥30 kg/m² | NR | NR | NR | NR | NR |
| Toye et al. [38] | Entire cohort  n=6 | Mean (SD): 67 (7) | 6 (100) | Mean (SD): 33 (3) | NR | NR | NR | NR | NR |
| **US** | | | | | | | | | |
| Buck et al. [42] | Entire cohort  n=338 | Mean (SD): 60.3 (10.8) | 51 (14) | Mean (SD): 33.2 (8.4) | Black or African American: 20 (6.4)  White: 267 (85.6)  Hispanic: 13 (4.2)  Asian: 2 (0.6)  Native Hawaiian: 0 (0.0)  Other: 2 (0.6)  Prefer not to answer: 7 (2.2) | NR | NR | NR | NR |
|  | BMI <30 kg/m2  n=124 | NR | NR | NR | NR | NR | NR | NR | NR |
|  | BMI ≥30 kg/m2  n=172 | NR | NR | NR | NR | NR | NR | NR | NR |
| Horn et al. [47] | Entire cohort  n=304 | Mean (SD): 58.1 (10.0) | 64 (21.1) | n (%) –  Obesity class I (BMI 30-<35): 103 (33.9)  Obesity class II (BMI 35-<40): 74 (24.3)  Obesity class III (BMI ≥40): 127 (41.8) | White: 265 (87.2)  Black/African American: 40 (13.2)  Spanish/Hispanic/Latino: 11 (3.6)  American Indian/Alaska Native: 9 (3.0) | n (%) –  Mild: 34 (11.2)  Moderate: 170 (55.9)  Severe: 100 (32.9) | NR | NR | NR |
| Miller et al. [32] | Entire cohort  n=11 | Mean (SD): 69.2 (4.9) | 4 (36) | Mean (SD): 29 (5.1) | NR | NR | NR | NR | NR |
| Seward, 2025 [58] | Entire cohort  n=60 | Mean (SD): 61 (NR) | 20 (33) | 44 (NR) | NR | NR | NR | NR | NR |
| Singh [35] | Entire cohort  n=48 | Mean (SD): 60.6 (9.8) | 12 (25) | NR | White: 22 (46)  African American: 26 (54) | NR | KOOS, mean (SD) –  Pain: 53.3 (47.2)  Symptoms: 49.3 (21.6)  Activities of daily living: 55.5 (19.9)  Sport/recreation: 34.2 (31.0)  Quality of life: 23.8 (22.5) | NR | Mean (SD): 7.8 (5.4) |
| Somers et al. [60] | Pain coping skills training only  n=60 | Mean (SD): 58.13 (11.25) | 20 (33) | Mean (95% CI): 34.4 (33.3-35.5) | Non-White: 23 (38) | KL score, mean (SD): 2.48 (1.01) | WOMAC pain, mean (95% CI): 42.8 (42.1-53.3) | NR | NR |
|  | Behavioral weight management only  n=59 | Mean (SD): 58.27 (11.02) | 12 (20) | Mean (95% CI): 33.5 (32.4-34.7) | Non-White: 20 (34) | KL score, mean (SD): 2.38 (1.12) | WOMAC pain, mean (95% CI): 42.6 (37.7-47.5) | NR | NR |
|  | Pain coping skills training and behavioral weight management  n=62 | Mean (SD): 57.47 (9.43) | 5 (8) | Mean (95% CI): 34.1 (33.0-35.2) | Non-White: 25 (40) | KL score, mean (SD): 2.38 (1.05) | WOMAC pain, mean (95% CI): 47.7 (42.1-53.3) | NR | NR |
|  | Standard care  n=51 | Mean (SD): 57.95 (10.41) | 11 (22) | Mean (95% CI): 34.1 (32.8-35.4) | Non-White: 20 (39) | KL score, mean (SD): 2.34 (1.05) | WOMAC pain, mean (95% CI): 43.4 (37.4-49.5) | NR | NR |
| Vincent 2023 [62] | Stable weight <25 kg/m2 | Mean (SD): 61.2 (9.3) | 74 (33.6) | NR | African American: 40 (18.2) Caucasian: 173 (78.6) Other: 7 (3.2) Hispanic: 4 (1.8) | NR | NR | KOOS-SFR: 85.3 (17.9) | NR |
|  | Stable weight 25 - 29.9 kg/m2 | Mean (SD): 63.3 (9.0) | 102 (49.5) | NR | African American: 36 (17.5) Caucasian: 166 (80.6) Other: 4 (1.9) Hispanic: 2 (0.9) | NR | NR | KOOS-SFR: 82.0 (21.4) | NR |
|  | Stabl;e weight >30kg/m2 | Mean (SD): 60.1 (8.5) | 45 (46.4) | NR | African American: 24 (24.7) Caucasian: 69 (71.2) Other: 3 (3.1) Hispanic: 1 (1.0) | NR | NR | KOOS-SFR: 77.5 (19.6) | NR |
|  | Steady weight loss | Mean (SD): 62.7 (9.3) | 7 (29.2) | NR | African American: 6 (25.0) Caucasian: 17 (70.8) Other: 1 (4.2) Hispanic: 0 (0) | NR | NR | KOOS-SFR: 61.6 (28.8) | NR |
|  | Steady weight gain | Mean (SD): 55.3 (7.0) | 10 (41.7) | NR | African American: 7 (9.2) Caucasian: 17 (70.8) Other: 0 (0) Hispanic: 0 (0) | NR | NR | KOOS-SFR: 76.7 (32.1) | NR |
|  | Cycling (Loss gain loss) | Mean (SD): 60.4 (9.0) | 40 (47.1) | NR | African American: 14 (16.5) Caucasian: 69 (81.2) Other: 2 (2.4) Hispanic: 0 (0) | NR | NR | KOOS-SFR: 74.3 (27.8) | NR |
|  | Cycling (Gain loss gain) | Mean (SD): 57.9 (8.2) | 26 (34.7) | NR | African American: 12 (16.0) Caucasian: 60 (80.0) Other: 3 (4.0) Hispanic: 2 (2.7) | NR | NR | KOOS-SFR: 76.6 (22.4) | NR |

Characteristics

Abbreviations: BMI = body mass index; GP = general practitioner; HOOS = hip osteoarthritis outcome score; KL = Kellgren-Lawrence; KOOS = knee injury and osteoarthritis outcome score; MUFIP = multifactorial intervention program; NR = not reported; NRS = numerical rating scale; OA = osteoarthritis; OKS = Oxford knee score; SF-8 = SF-8 Health Survey; TUG = Timed Up and Go; VAS = visual analog scale; WOMAC = Western Ontario and McMaster universities osteoarthritis index.

**Supplementary Table 3**. Summary of Healthcare Professionals’ Characteristics from Included Studies

| **Author, Year** | **Study Arm/ Sample Size** | **Age (years)** | **Male, n (%)** | **Healthcare Role** | **Experience (years)** |
| --- | --- | --- | --- | --- | --- |
| **Australia** | | | | | |
| Allison et al. [19] | Entire cohort (n=13) | Mean (SD): 37.7 (9.8) | 5 (38.5) | Physical therapist | Mean (SD): 15.3 (10.5) |
| Allison, 2025 [18] | Entire cohort (n=6) | NR | 4 (66.7) | Physical therapists (aka physiotherapists) | Median clinical experience: 3.5 (range 2.8 - 28.3) years |
| Egerton et al. [23] | Entire cohort (n=11) | NR | 4 (36.4) | General practitioner | Mean (SD): 22 (13) |
| Lawford et al. [30] | Entire cohort (n=5) | NR | NR | Dietitian | Mean (SD): 8.5 (6.2) |
| Nguyen, 2025 [33] | Entire cohort (n=30) | Mean (SD): 36.9 (11.1) | 17 (57) | Physical therapist | Mean (SD): 11.9 (9.4) |
| Wallis et al. [39] | Entire cohort (n=35) | NR | 25 (71) | n (%)  Orthopedic surgeon: 9 (26)  Rehabilitation physician: 9 (26)  General practitioner: 5 (14)  Rheumatologist: 5 (14)  Sports physician: 5 (14)  Emergency physician: 2 (6) | n (%) –  Less than 5 years: 3 (9)  6-10 years: 7 (20)  11-15 years: 8 (23)  16-20 years: 9 (25)  21-25 years: 2 (5)  26-30 years: 3 (9)  31 years and over: 3 (9) |
| **Canada** | | | | | |
| MacKay et al. [31] | Entire cohort (n=33) | NR | 8 (24) | Physical therapist | Mean (range): 21 (1-45) |
| **The Netherlands** | | | | | |
| Knoop et al. [27] | Entire cohort (n=16) | Mean (SD): 41 (14) | 6 (38) | n (%)  Physiotherapist: 11 (69)  Dietitian: 5 (31) | Mean (SD): 17 (14) |
| **Portugal** |  |  |  |  |  |
| Ferreira et al. 2023 [24] | E-survey  n=120  Semi-structured interviews  N=10 | E-survey  Age category, n (%): 20-29 years 34 (28.3%) 30-39 years 54 (45%) 40-49 years 13 (10.8%) ≥ 50 years 19 (15.8%)  Semi-structured interviews  Age category, n (%): 20-29 years 34 (40%) 30-39 years 54 (40%) 40-49 years 13 (10%) < 50 years 19 (10%) | E-survey 36 (30) Semi-structured interviews 4 (40) | Physiotherapists | E-survey (n=120)  > 15 38 (31.7%)  < 5 years 18 (15%)  5-10 years 41 (34.2%)  11-15 years 30 (25%)  > 15 years 31 (25.8%)  Semi-structured interviews (n=10)  > 5 3 (33.3%)  < 15 years 2 (20%)  5-10 years 3 (33.3%)  11-15 years 2 (20%)  > 15 years 0(0%) |
| **Saudi Arabia** |  |  |  |  |  |
| Mal et al. [50] | Entire cohort (n=373) | Mean (SD): 31.25 (7.17) | 198 (52.4) | Physical therapists | <6 years of clinical experience: 174 (46%)  6–10 years of clinical experience: 105 (27.8)  >10 years of clinical experience: 99 (26.2) |
| **Singapore** |  |  |  |  |  |
| Tan et al. [36] | Dietetics and Nutrition (NND) component of the CONNACT intervention (n=14) | NR | 3 (13.6) | Psychologist: 1 (4.5)  Medical social worker: 1 (4.5)  Dietician: 2 (9.1)  Physiotherapist: 5 (22.7)  Doctors: 5 (22.7) | NR |
| **United Kingdom** | | | | | |
| Hill et al. [46] | Entire cohort (n=205) | NR | NR | Surgeon | NR |
| Hill et al. [45] | Entire cohort (n=130) | NR | NR | General practitioner | NR |
| Simpson, 2024 [59] | Weight loss component of package-of-care intervention (4 qualitative research nurse interviews and 2 surgeon interviews) | NR | NR | Research nurse | NR |
| **US** | | | | | |
| Buck et al. [42] | Entire cohort (n=120) | NR | NR | n (%)  Primary Care or Subspecialty Physician: 32 (27)  Nurse Practitioner or Physician Assistant: 1 (1)  Rehabilitation Professional (physical therapist, occupational therapist, or certified athletic trainer): 67 (56)  Nutrition Professional, Exercise Professional, or Registered Nurse: 12 (10)  Other: 8 (7) | NR |
| Godziuk, 2023 [25] | Entire cohort (n-45) | Mean (SD): 54.3 (12.4) | 40 (89) | Orthopedic surgeon | Surgical experience: 21.2 (13.3) years (range, 2 to 44 years) |
| Horn et al. [47] | Entire cohort (n=101) | NR | 74 (73) | Primary care physician | Mean (SD): 19.4 (8.5) |
| Miller et al. [32] | Entire cohort (n=6) | NR | NR | Physician | NR |

Abbreviations: HCP = healthcare professional; KOA = osteoarthritis of the knee; NR = not reported

**Supplementary Table 4.** Quality Assessment of Cohort and Cross-sectional Studies

| **Author, Year** | **Selection** | **Comparability** | **Outcome** | **Total score** |
| --- | --- | --- | --- | --- |
| **Cohort Studies** | | | | |
| Aree-Ue et al. [61] | 2 | 0 | 1 | 3 |
| Vincent et al. [62] | 3 | 2 | 3 | 8 |
| **Cross-Sectional Studies** | | | | |
| Baumbach et al. [40] | 2 | 2 | 1 | 5 |
| Bopf et al. [41] | 2 | 0 | 1 | 3 |
| Buck et al. [42] | 2 | 0 | 1 | 3 |
| Ekram et al. [43] | 1 | 0 | 1 | 2 |
| Grønhaug et al. [44] | 2 | 0 | 1 | 3 |
| Hill et al. [46] | 1 | 0 | 1 | 2 |
| Hill et al. [45] | 1 | 0 | 1 | 2 |
| Horn et al. [47] | 1 | 0 | 1 | 2 |
| Howarth et al. [48] | 2 | 0 | 1 | 3 |
| Ingelsrud et al. [49] | 2 | 0 | 1 | 3 |
| Mal et al. [50] | 2 | 0 | 2 | 4 |
| Oomen et al. [51] | 2 | 0 | 1 | 3 |
| Østerås et al. [52] | 2 | 0 | 2 | 4 |
| Richette et al. [53] | 3 | 0 | 2 | 5 |
| Siddique et al. [54] | 3 | 0 | 2 | 5 |

Note: Quality assessment was conducted using the Newcastle-Ottawa Scale, which includes the following three main components: (a) selection, which evaluates the adequacy of the study groups selection (scored from 0-4), (b) comparability, which assesses the comparability of the study groups (scored from 0-2); and (c) outcome, which evaluates the outcome assessment quality (scored from 0-3). The total score ranges from 0-9, with higher scores indicating higher quality.

**Supplementary Table 5.** Quality Assessment of Qualitative Studies

| **Author, Year** | **Was there a clear statement of the aims of the research?** | **Is a qualitative methodology appropriate?** | **Was the research design appropriate to address the aims of the research?** | **Was the recruitment strategy appropriate to the aims of the research?** | **Was the data collected in a way that addressed the research issue?** | **Has the relationship between researcher and participants been adequately considered?** | **Have ethical issues been taken into consideration?** | **Was the data analysis sufficiently rigorous?** | **Is there a clear statement of findings?** | **How valuable is the research?** |
| --- | --- | --- | --- | --- | --- | --- | --- | --- | --- | --- |
| Allison et al., 2019 [17] | Yes | Yes | Yes | Yes | Yes | Can't Tell | Yes | Yes | Yes | Yes |
| Allison et al., 2019 [19] | Yes | Yes | Yes | Yes | Yes | Can't Tell | Yes | Yes | Yes | Yes |
| Allison et al., 2025 [18] | Yes | Yes | Yes | Yes | Yes | Can't Tell | Yes | Yes | Yes | Yes |
| Bunzli et al., 2019 [20] | Yes | Yes | Yes | Yes | Yes | Can't Tell | Yes | Yes | Yes | Yes |
| Carmona-Teres et al., 2017 [21] | Yes | Yes | Yes | Yes | Yes | Can't Tell | Yes | Yes | Yes | Yes |
| Coetzee et al., 2020 [22] | Yes | Yes | Yes | Yes | Yes | Can't Tell | Yes | Yes | Yes | Yes |
| Egerton et al., 2018 [23] | Yes | Yes | Yes | Yes | Yes | Can't Tell | Yes | Yes | Yes | Yes |
| Ferreira et al., 2023 [24] | Yes | Yes | Yes | Yes | Yes | Can't Tell | Yes | Yes | Yes | Yes |
| Godziuk et al., 2023 [25] | Yes | Yes | Yes | Yes | Yes | Can't Tell | Yes | Yes | Yes | Yes |
| Isla Pera et al., 2016 [26] | Yes | Yes | Yes | Yes | Yes | Can't Tell | Yes | Yes | Yes | Yes |
| Knoop et al., 2022 [27] | Yes | Yes | Yes | Yes | Yes | Can't Tell | Yes | Yes | Yes | Yes |
| Lawford et al., 2021 [30] | Yes | Yes | Yes | Yes | Yes | Can't Tell | Yes | Yes | Yes | Yes |
| Lawford et al., 2023 [28] | Yes | Yes | Yes | Yes | Yes | Can't Tell | Yes | Yes | Yes | Yes |
| Lawford et al., 2024 [29] | Yes | Yes | Yes | Yes | Yes | Can't Tell | Yes | Yes | Yes | Yes |
| MacKay et al., 2020 [31] | Yes | Yes | Yes | Yes | Yes | Can't Tell | Yes | Yes | Yes | Yes |
| Miller et al., 2020 [32] | Yes | Yes | Yes | Yes | Yes | Can't Tell | Yes | Yes | Yes | Yes |
| Nguyen et al., 2025 [33] | Yes | Yes | Yes | Yes | Yes | Can't Tell | Yes | Yes | Yes | Yes |
| Pavlovic et al., 2023 [34] | Yes | No | Yes | Yes | Yes | Can't Tell | Yes | Yes | Yes | Yes |
| Singh et al., 2021 [35] | Yes | Yes | Yes | Yes | Yes | Can't Tell | Yes | Yes | Yes | Yes |
| Tan et al., 2025 [37] | Yes | Yes | Yes | Yes | Yes | Can't Tell | Yes | Yes | Yes | Yes |
| Tan et al. [36] | Yes | Yes | Yes | Yes | Yes | Can't Tell | Yes | Yes | Yes | Yes |
| Toye et al., 2018 [38] | Yes | Yes | Yes | Yes | Yes | Can't Tell | Yes | Yes | Yes | Yes |
| Wallis et al. [39] | Yes | Yes | Yes | Yes | Yes | Can't Tell | Yes | Yes | Yes | Yes |

**Supplementary Table 6.** Quality Assessment of Randomized Controlled Trials

| **Author, Year** | **Randomization process** | **Deviations from intended interventions** | **Missing outcome data** | **Measurement of the outcome** | **Selection of the reported result** | **Overall Bias** |
| --- | --- | --- | --- | --- | --- | --- |
| Allison et al. [55] | Low risk | Low risk | Low risk | High risk | Low risk | Low risk |
| Bennell et al. [56] | Low risk | Low risk | Low risk | High risk | Some concerns | Some concerns |
| Messier et al. [57] | High risk | Low risk | Low risk | High risk | Low risk | Some concerns |
| Seward et al. [58] | Low risk | Low risk | High risk | High risk | High risk | High risk |
| Simpson et al. [59] | Low risk | Low risk | Low risk | High risk | Low risk | Low risk |
| Somers et al. [60] | Low risk | Some concerns | Low risk | High risk | Some concerns | Some concerns |

Note: Quality assessment was conducted using the Cochrane risk-of-bias tool for randomized controlled trials
